# A commentary on “Acupuncture for rehabilitation after total knee arthroplasty: a systematic review and network meta-analysis”

**DOI:** 10.1097/JS9.0000000000004609

**Published:** 2026-03-03

**Authors:** Qiang Liu, Cui Liu, Xin Qin

**Affiliations:** a Gansu University of Chinese Medicine, Lanzhou, Gansu, China; b Gansu Provincial Second People’s Hospital, Lanzhou, Gansu, China

*Dear Editor,*

We read with great interest the network meta-analysis by Li *et al*^[1]^, “Acupuncture for rehabilitation after total knee arthroplasty: a systematic review and network meta-analysis,” published in the International Journal of Surgery. This study synthesized 28 randomized controlled trials (*n* = 2128) and found that electroacupuncture was associated with superior analgesic effects, while auricular acupressure showed favorable signals for motor recovery, with adverse events generally mild. The Visual Analogue Scale (VAS) network appeared well connected and centered on electroacupuncture, whereas funnel-plot asymmetry suggested possible small-study effects. We commend the authors for this valuable contribution and, building on their findings, wish to offer three perspectives that may further enhance clinical applicability and translational relevance. This correspondence also adheres to the TITAN Guidelines 2025 for the declaration and use of artificial intelligence in scientific manuscripts^[2]^.

Across the included trials, parameters for electroacupuncture (EA) varied substantially (e.g., stimulation frequencies ranging from 2 to 100 Hz), and interventions were initiated pre-, intra-, or post-operatively – factors that plausibly influence treatment effects yet were not modeled explicitly. We recommend re-extracting intervention details in accordance with STRICTA/TIDieR (frequency, intensity, per-session and total duration, number of sessions, and start time relative to surgery) to derive a cumulative stimulation dose (CSD) and a standardized timing window (pre/intra/post). These covariates can then be incorporated into meta-regression or covariate-adjusted NMA to delineate dose–response and time-window–response relationships, shifting the emphasis from “which technique ranks first” to “which parameterization works best, and when.” This approach is consistent with the updated CONSORT 2025 guidance emphasizing complete, transparent reporting for randomized trials, which strengthens reproducibility and enables robust secondary analyses^[3]^.

Classifying “EA, auricular acupressure, warm acupuncture, laser acupuncture, dry needling,” and related modalities as mutually exclusive trial arms risks conflating a base needling effect with add-on components (e.g., electrical stimulation, laser, heat, transcutaneous stimulation). A component network meta-analysis (CNMA) framework can encode “base acupuncture” plus enhancers as distinct components to estimate their marginal and interaction effects and to address pragmatic questions such as: How much incremental benefit does electrical stimulation confer when added to base acupuncture within multimodal rehabilitation? Contemporary clinical CNMAs – for example, a 2024 analysis of CBT-I – demonstrate how component-resolved evidence can guide streamlined, high-yield intervention packages, an approach directly applicable here^[4]^.

In the index NMA, VAS served as the primary endpoint and HSS/ROM/WOMAC (stiffness) as secondary outcomes. While clinically informative, these measures do not fully capture priorities within Enhanced Recovery After Surgery (ERAS) pathways: opioid consumption (MME), PONV and delirium, time to first ambulation, length of stay (LOS), 30-day readmission, and costs. We therefore recommend harmonizing analytic timepoints (e.g., 0–48 h, pre-discharge, 6–12 weeks) and incorporating these value-based endpoints into an updated synthesis. Recent high-quality evidence from JAMA Network Open shows ERAS programs reduce LOS and early complications across randomized trials, supporting endpoint sets that resonate with clinical operations and payers^[5]^.

In conclusion, this rigorous NMA offers valuable evidence on acupuncture after TKA. To enhance clinical and policy relevance, we respectfully recommend three refinements: parameterized EA reporting and analysis (cumulative stimulation dose and standardized timing) to enable dose- and time-response modeling; use of component NMA to separate base needling from add-on components and quantify their effects; and ERAS-aligned endpoints with harmonized timepoints to capture pathway-level value. We appreciate the authors’ contribution and look forward to further advances.

## Data Availability

This commentary does not involve original data or code generation. All discussions are based on published findings and publicly available literature.

## TITAN checklist

A completed TITAN 2025 checklist is provided as a Supplemental Digital Content File, available at: http://links.lww.com/JS9/G770.
